# Inhibition of the cell migration, invasion and chemoresistance of colorectal cancer cells through targeting KLF3 by miR-365a-3p

**DOI:** 10.7150/jca.61967

**Published:** 2021-08-26

**Authors:** Jing Li, Rubing Mo, Linmei Zheng

**Affiliations:** 1 Department of Emergency Surgery, Hainan General Hospital, Hainan Affiliated hospital of Hainan medical university, Haikou, Hainan Province, 570311, China.; 2 Department of Pneumology, Hainan General Hospital, Hainan Affiliated hospital of Hainan medical university, Haikou, Hainan Province, 570311, China.; 3 Department of Obstetrics, Hainan General Hospital, Hainan Affiliated hospital of Hainan medical university, Haikou, Hainan Province, 570311, China.

**Keywords:** Colorectal cancer, Chemo-resistance, MicroRNAs, KLF3, Migration, Invasion

## Abstract

**Background:** Metastasis and chemoresistance limit treatment efficacy of colorectal cancer (CRC) patients. MicroRNAs (miRNAs) have been believed to be candidate biomarkers for tumor cell proliferation, metastasis and chemoresistance, but the related molecular mechanisms are not clear for prognosis prediction.

**Aims:** We aimed to investigate the role of miR-365a-3p in metastasis and chemoresistance of CRC.

**Methods:** The expression levels of miR-365a-3p in clinical CRC tissues were analyzed. The effects of miR-365a-3p expression levels on tumor chemoresistance, invasion and migration were also determined. A dual luciferase reporter gene assay was used to determine the effect of miR-365a-3p on its target gene, Kruppel-like factor 3 (KLF3), and the effect of the miR-365a-3p/KLF3 axis on CRC cell chemoresistance, migration and invasion was further investigated.

**Results:** In patients with CRC with lymph node or distant organ metastasis or in CRC cell lines, the expression levels of miR-365a-3p were significantly downregulated. In addition, the findings of Transwell assays demonstrated that miR-365a-3p significantly suppressed CRC cell migration and invasion. The dual luciferase reporter gene assay results suggested that miR-365a-3p may play an important role in the regulation of migration, invasion and chemoresistance in CRC cells.

**Conclusions:** The findings of present study provided evidence to suggest that miR-365a-3p may be a potential tumor suppressor gene in CRC and may inhibit the migration, invasion and chemoresistance of CRC cells. These results suggested that targeting miR-365a-3p/KLF3 axis may represent a potential therapeutic intervention for metastatic disease in patients with CRC.

## Introduction

Colorectal cancer (CRC) is the third most common type of cancer worldwide. Surgical resection, combined with chemotherapy, is the primary therapeutic option for patients with CRC 1. Nevertheless, metastasis and chemoresistance remain a challenge for effective CRC disease control and improving the overall prognosis of patients with CRC. Due to complex genetic and molecular background of CRC, the exact underlying mechanism of chemoresistance remains to be determined. Several previous studies have reported that the dysfunction of several signaling pathways was associated with CRC chemoresistance 2, 3. For example, the dysregulation of Wnt/β-catenin signaling pathway was demonstrated to be associated with tumor cell proliferation and inhibition of differentiation 4. In addition, the dysfunction of NF-κB 5, PI3K/AKT 6 and RAS/RAF/MEK/MAPK 7 signaling axes was associated with enhanced tumor cell proliferation and suppressed cellular apoptosis. Therefore, a more in-depth understanding of the detailed molecular regulatory network of CRC is required to develop effective strategies for the treatment of metastatic disease.

MicroRNAs (miRNAs/miRs) are a large family of non-coding RNAs 8 that have been discovered to play important roles in cancer progression and metastasis 9. Previous studies have identified several miRNAs that played significant roles in CRC progression and metastasis 10. For example, the expression levels of miR-92 and miR-17-3p were found to be significantly upregulated in CRC samples. Notably, the expression levels of these miRNAs were markedly downregulated following surgical treatment, indicating their potential role on tumor burden and application for prognostic evaluation 11. In another previous study, the upregulated expression levels of miR-141 in the plasma of 102 patients with stage IV CRC was also found to predict poor survival 12. In addition, the expression of miR-141 was upregulated in CRC metastasis patients (M1 stage) compared with M0 stage patients. Patients with CRC with distant metastases, lymph node involvement or an advanced tumor stage were also found to have upregulated expression levels of miR-183 13. Furthermore, miR-182 was also shown to be positively associated with tumor size and lymph node metastasis in another study 14.

The involvement of miR-365a-3p in several kinds of tumors has been reported. miR-365a-3p could suppress the gastric cancer growth through targeting HELLS/GLUT1 Axis 15. miR-365a-3p could inhibit proliferation and invasion of Hep-2 cells through targeting ten-eleven translocation 1 16. miR-365a-5p suppressed gefitinib resistance in non-small-cell lung cancer through targeting PELI3 17. However, the regulatory role of miR-365a-3p in the CRC has not been fully investigated.

Nonetheless, despite current advances in molecular research on the role of miRNAs in the early diagnosis of CRC, the understanding of the significance of miRNAs in metastatic disease prediction of patients with CRC remains limited. Previous studies have indicated that the upregulated expression of several miRNAs was associated with metastatic disease in patients with CRC 18, 19. Thus, further investigations are required to identify biomarkers that can predict the occurrence of cancer metastasis.

The present study used CRC clinical samples and cell lines to determine the role of miR-365a-3p in CRC migration, invasion and chemoresistance. Further studies were performed to identify potential target genes of miR-365a-3p that could potentially influence the migration, invasion and chemoresistance of CRC cells. This study firstly demonstrated the inhibition role of miR-365a-3p in the cell migration, invasion and chemoresistance of CRC through KLF3. These findings might provide potential novel therapeutic strategies for the prevention and treatment of CRC.

## Material and methods

### Patient studies

A total of 162 patients diagnosed with CRC were recruited from Hainan General Hospital between February 2019 and August 2020, and underwent surgical resection to obtain CRC and adjacent normal tissue. The patients had not received any type of treatment prior to surgery. Tissues were stored in liquid nitrogen post-resection until required for further analysis. The present study was approved by the Ethics Committee of Hainan General Hospital Cancer Center (Approval number: 2019-068), and written informed consent was obtained from all patients prior to participation. The detailed clinic parameters of enrolled patients have been listed in Table 1. The inclusion criteria of enrolled patients were listed as follows: The CRC patients confirmed via biopsy and histological testing. The exclusion criteria were listed as follows: (1) additional gastrointestinal disorders (e.g., Crohn's disease or ulcerative colitis); (2) clinically significant immunodeficiency; (3) patient was pregnant.

### Cell lines and culture

CRC cell lines (HCT8, DLD1, LoVo, SW48, HT29, SW480, RKO and HCT116) and a human normal fetal colonic mucosa cell line (FHC) were obtained from the American Type Culture Collection (ATCC, Manassas, VA, USA). Cells were cultured in RPMI-1640 medium supplemented with 10% FBS (Hyclone; Cytiva, USA), 100 IU/ml penicillin and 100 μg/ml streptomycin (Invitrogen, Carlsbad, CA, USA), and maintained at 37 °C with 5% CO_2_.

### Lentiviral vector construction and transfection

The lentiviral pLV-THM expression vector containing a cytomegalovirus-driven enhanced green florescence protein and H_1_ promoter upstream of *Cla*I and *Mlu*I restriction sites was purchased from Clontech Laboratories, Inc. The miR-365a-3p mimic and miR-365a-3p inhibitor sequences were inserted between *Cla*I and *Mlu*I sites of pLV-THM plasmid. PCR was subsequently conducted using DNA templates and miR-365a-3p mimic and inhibitor sequences, and the PCR products were purified by 1% agarose gel electrophoresis and double digested with *Bam*HI and *Eco*RI alongside empty pLVX vectors. The ligation reaction between the vector and purified PCR products was performed overnight using T4DNA ligase, and the ligation product was further used for the DH5α competent *Escherichia coli* cell transformation. The cells were then plated into an ampicillin-containing LB plate and incubated at 37 °C overnight. Positive clones were collected and the plasmid was sequenced after extraction (Invitrogen, Carlsbad, CA, USA).

Lentiviral vectors carrying miR-365a-3p mimic, miR-365a-3p inhibitor and negative control (NC; empty vector) were transfected into CRC cells with a multiplicity of infection of 20 (after packaging) and a vector titer setting of 1×10^9^ cells/ml. The fluorescent protein expression levels were evaluated and transfection efficiency was determined following 24-48 h of transfection.

### Reverse transcription-quantitative PCR (RT-qPCR)

Total RNA was extracted using TRIzol^®^ reagent (Invitrogen, Carlsbad, CA, USA). Total RNA was reverse transcribed into cDNA and qPCR was subsequently performed. The following thermocycling conditions were used for the qPCR: 40 cycles at 94 °C for 30 sec, 55 °C for 30 sec and 72 °C for 90 sec. The following primers pairs were used for qPCR: Kruppel-like factor 3 (KLF3) forward, 5'-CTCATGGTCTCCTTATCGGAGG-3' and reverse, 5'-TGTCCTCTGTGGTTCGATCCCA-3'; U6 forward, 5'- GACTATCATATGCTTACCGT-3' and reverse, 5'-AACGCTTCACGAATTTGCGT-3'; miR-365a-3p forward, 5'-GCCCCTAAAAATCCTT-3' and reverse, 5'- GTGCAGGGTCCGAGGT-3'; and β-actin forward, 5'- CACCATTGGCAATGAGCGGTTC-3' and reverse, 5'- AGGTCTTTGCGGATGTCCACGT-3'.

### Western blotting

Total protein was extracted from tissues or cells using RIPA lysis buffer (Beyotime Institute of Biotechnology, Shanghai, China). Total protein was quantified using a BCA protein assay kit (Beyotime Institute of Biotechnology, Shanghai, China) and 30 μg protein/lane was separated via 10% SDS-PAGE. The separated proteins were subsequently transferred onto PVDF membranes (EMD Millipore, Bedford, MA, USA,) and blocked with TBS containing 5% skimmed milk for 2 h at room temperature. The membranes were then incubated with an anti-KLF3 primary antibody (Abcam; cat. no. ab154531; 1:1,000, Cambridge, UK) at 4 °C overnight. Following primary antibody incubation, the membranes were rinsed with TBS-Tween 20 buffer solution (Sigma-Aldrich; Merck KGaA) and incubated with a secondary antibody (Abcam; cat. no. ab205718; 1:2,000, Cambridge, UK) for 1 h at room temperature. Protein bands were visualized using enhanced chemiluminescence and densitometric analysis was performed using Quantity One software (Bio-Rad Laboratories, Inc, CA, USA).

### Flow cytometric analysis of apoptosis

Cells were treated with different concentrations of fluorouracil (0, 10, 20, 30, 40, 50, 60, 70 or 80 g/ml), cisplatin (0, 5, 10, 15, 20, 25, 30, 35 or 40 µM) or doxorubicin (0, 0.25, 0.5, 1, 2, 4, 6 or 12 µM) for 4 h, then washed with PBS and fixed overnight in 75% ethanol at -20 °C. Following overnight incubation, the cells were incubated with Annexin V-FITC (Thermo, Waltham, Massachusetts, USA) and propidium iodide (Sigma-Aldrich, St. Louis, Missouri, USA) for 20 min at room temperature. Apoptotic cells were analyzed using flow cytometry (FACScan™; BD Biosciences, San Jose, California, USA).

### Transwell assay

Cells were treated as described for flow cytometric analysis. Then, 5×10^4^ transfected cells were plated into the upper chamber of a Transwell plate (pore size, 8-μm; Corning, Inc.). The lower chambers were filled with medium supplemented with 10% FBS (Gibco, Langley, OK, USA). Following incubation for 48 h at 37 °C with 5% CO_2_, the cells were fixed with 70% ethanol for 30 min and stained with 0.1% crystal violet for 20 min. The number of cells in the lower chamber was counted in 10 randomly selected fields of view/sample using a microscope (Leica Microsystems GmbH).

### Dual luciferase reporter assay

After digestion with trypsin, 1×10^5^ SW480 and LOVO cells were plated into 24-well plates prior to transfection. Wild-type (WT) or mutant (MUT) 3'-untranslated region (UTR) sequences of KLF3 were cloned into plasmids. Subsequently, cells were transfected with either WT- or MUT-KLF3 3'-UTR plasmids and miR-NC or miR-365a-3p inhibitor/mimic using transfection reagent diluted in culture medium for 48 h. The transfection efficiencies were evaluated using a fluorescence microscope (Leica Microsystems GmbH).

### Statistical analysis

Statistical analysis was performed using SPSS 22.0 software (IBM Corp.), and GraphPad Prism version X software (GraphPad Software, Inc.) was used to generate the figures. Statistical differences between two groups were determined using a Student's t-test. Correlation analysis was performed using Pearson's correlation analysis, and a χ^2^ test was used to analyze classification data. P<0.05 was considered to indicate a statistically significant difference.

## Results

### Expression levels of miR-365a-3p are downregulated in CRC tissues and cell lines

**Figure 1:**
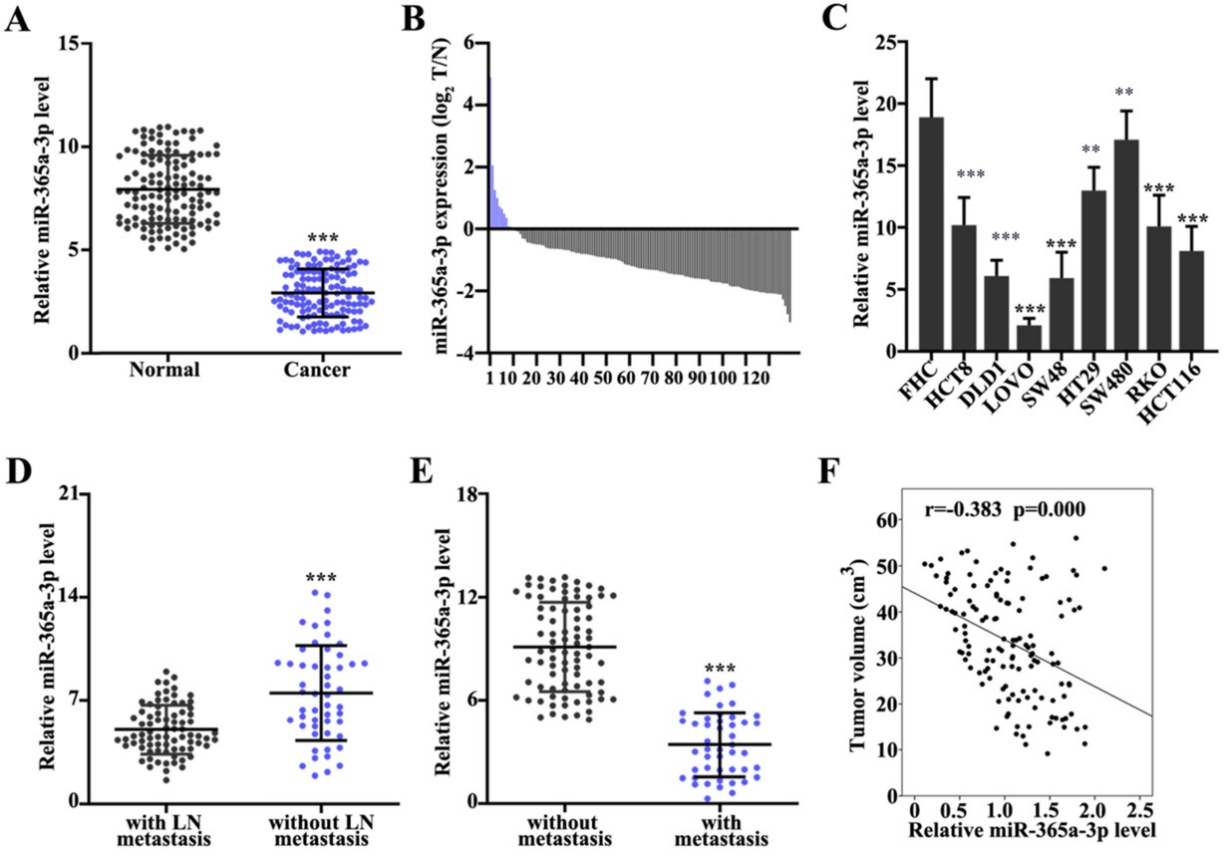
** Expression levels of miR-365a-3p are downregulated in CRC tissues and cell lines. (A)** RT-qPCR was used to analyze miR-365a-3p expression levels in 162 CRC and adjacent normal tissues. **(B)** Log2 transformed value of miR-365a-3p expression level ratio between CRC and adjacent normal tissues. **(C)** RT-qPCR was used to determine miR-365a-3p expression levels in different CRC cell lines (HCT8, DLD1, LoVo, SW48, HT29, SW480, RKO and HCT116) and a human normal fetal colonic mucosa cell line (FHC). RT-qPCR was performed to analyze miR-365a-3p expression levels in CRC samples with or without **(D)** lymph node metastasis or **(E)** distant organ metastasis. **(F)** Association between relative miR-365a-3p expression levels in CRC tumor tissue and tumor volume of surgically resected clinical samples. RT-qPCR, reverse transcription-quantitative PCR; miR, microRNA; CRC, colorectal cancer. **P<0.05 compared with group FHC, *** P<0.01 compared with group FHC.

First, the expression levels of miR-365a-3p in CRC and adjacent normal tissues were analyzed. The results demonstrated that the expression of miR-365a-3p were significantly downregulated in CRC tissues compared with adjacent normal tissues (Fig. 1A). The miR-365a-3p expression level ratio between CRC and adjacent normal tissues also revealed that the majority of tumor samples had significantly downregulated expression levels of miR-365a-3p compared with adjacent normal tissues (Fig. 1B). To validate these findings, the expression levels of miR-365a-3p were also investigated in different CRC cell lines; the data revealed that miR-365a-3p expression levels were significantly downregulated in CRC cell lines (HCT8, DLD1, LOVO, SW48, HT29, SW480, RKO and HCT116) compared with the human normal fetal colonic mucosa cell line, FHC (Fig. 1C), which was consistent with the results obtained from the patient samples. In addition, the expression levels of miR-365a-3p in patients with CRC with or without lymph node or distant organ metastasis were further investigated. The expression levels of miR-365a-5p were significantly downregulated in patients with CRC with lymph node or distant organ metastasis compared with patients without metastasis (Fig. 1D and E). In addition, miR-365a-3p expression was found to be negatively associated with CRC tumor volume (P<0.001; Fig. 1F).

### Effect of miR-365a-3p on migration and invasion of CRC cell lines

**Figure 2:**
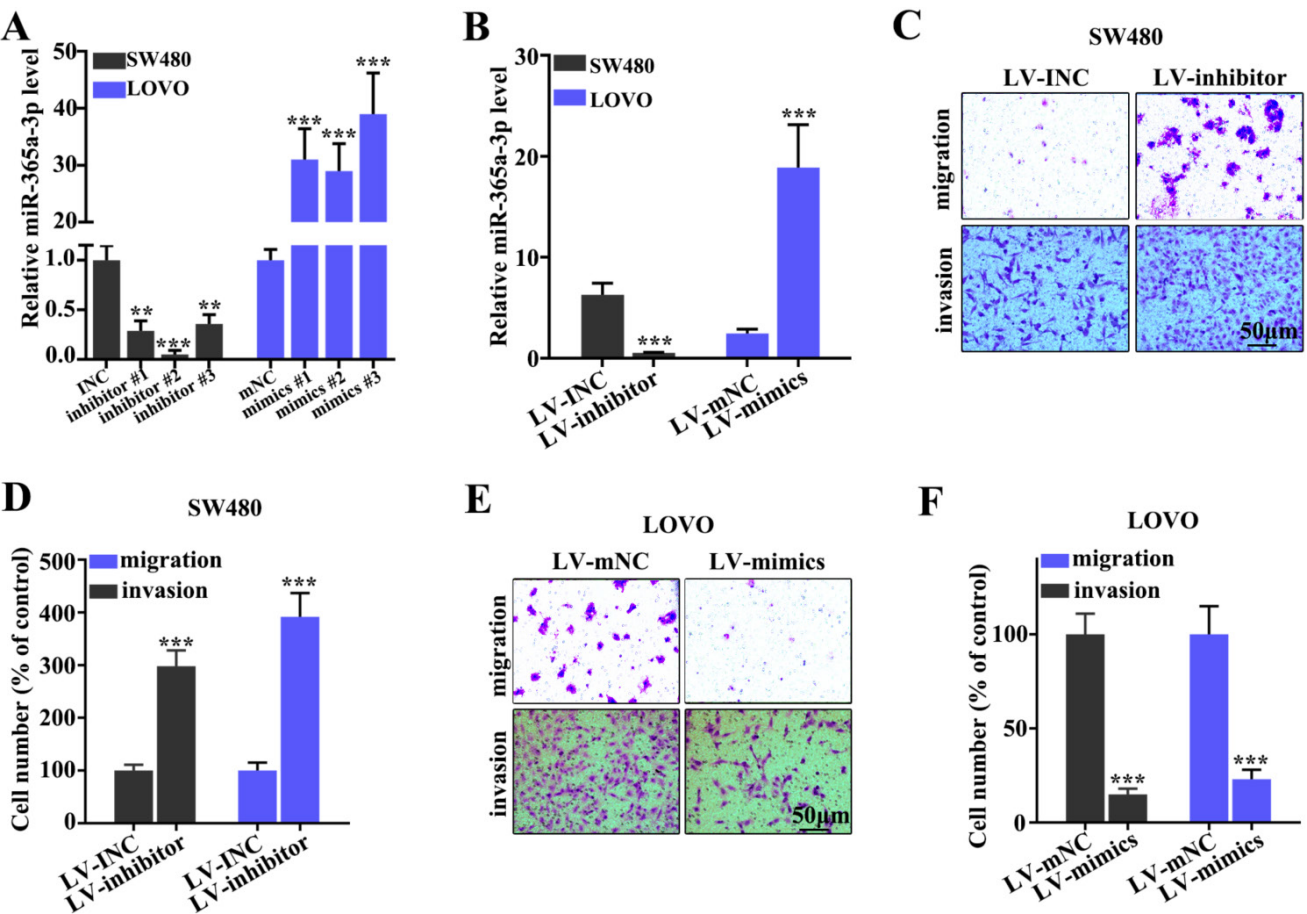
** Effect of miR-365a-3p on the migration and invasion of CRC cell lines. (A)** RT-qPCR was used to analyze miR-365a-3p expression levels in SW480 and LOVO cells transfected with three different miR-365a-3p-specific inhibitors or mimics, respectively. **(B)** RT-qPCR was used to determine miR-365a-3p expression levels in SW480 and LOVO cells transfected with lentiviral vectors carrying a miR-365a-3p-specific inhibitor or mimic, respectively. **(C-F)** Migratory and invasive abilities of SW480 and LOVO cells transfected with lentiviral vectors carrying a miR-365a-3p-specific inhibitor or mimic, respectively, were determined. RT-qPCR, reverse transcription-quantitative PCR; miR, microRNA. **P<0.05 compared with group INC, mNC, LV-INC, or LV-mNC. *** P<0.01 compared with group INC, mNC, LV-INC, or LV-mNC.

To determine the effect of miR-365a-3p on migration and invasion of CRC cell lines, three different miR-365a-3p-specific inhibitors and mimics were designed (Fig. 2A). As shown in Fig. 2B, transfection with three miR-365a-3p-specific inhibitors and mimics significantly downregulated and upregulated the expression levels of miR-365a-3p, respectively. Lentiviral vectors carrying the miR-365a-3p inhibitor were constructed and transfected into SW480 cell line, while lentiviral vectors carrying the miR-365a-3p mimic were constructed and transfected into LOVO cell line. These plasmids also exhibited significant regulatory effects on miR-365a-3p expression. The effect of miR-365a-3p modulation on migration and invasion of SW480 and LOVO cell lines was subsequently investigated. In cells transfected with lentiviral vector carrying the miR-365a-3p inhibitor, the invasive and migratory abilities were significantly increased (Fig. 2C-F). Conversely, in cells transfected with the lentiviral vector carrying the miR-365a-3p mimic, the migration and invasion were significantly suppressed (Fig. 2C-F).

### Interference of miR-365a-3p expression alters the viability of CRC cells following treatment with chemotherapy agents

**Figure 3:**
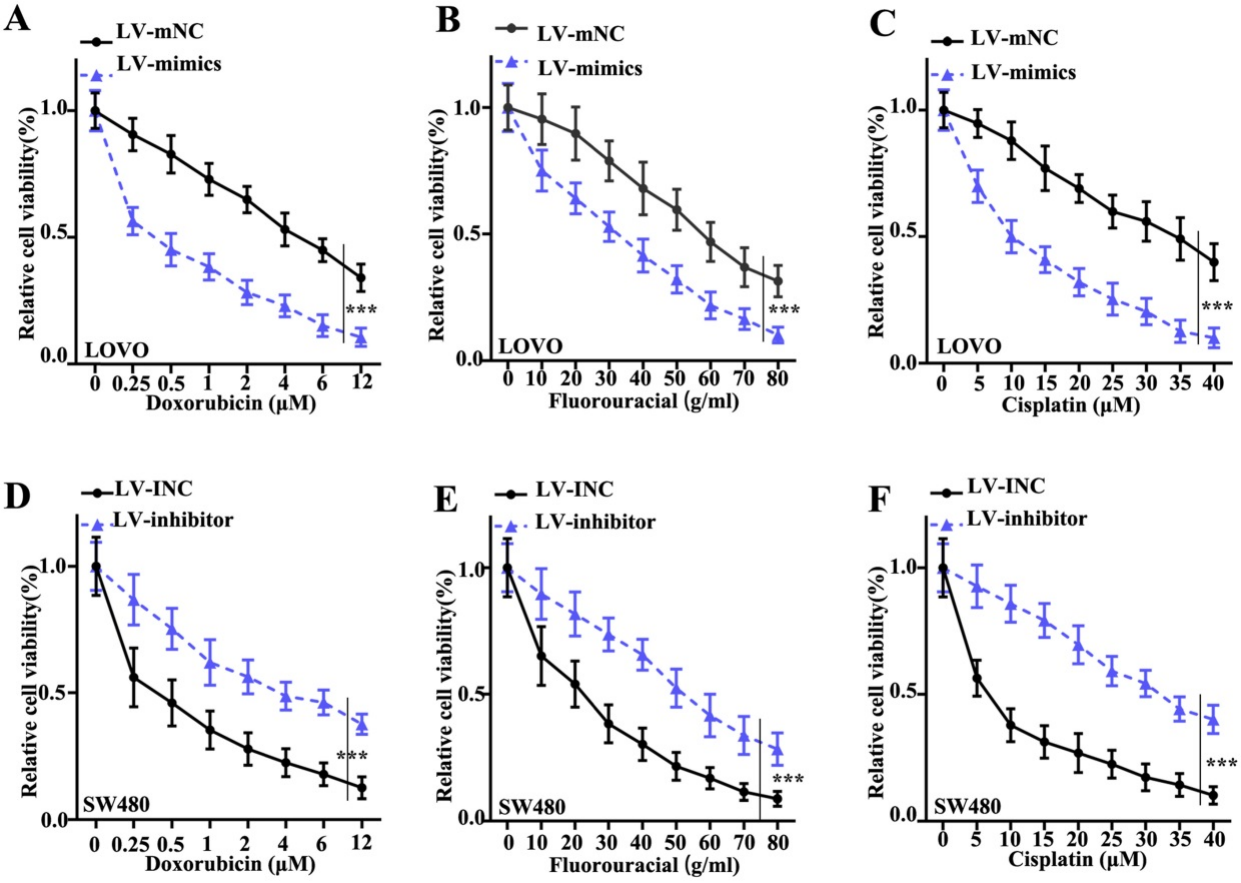
** Interference of miR-365a-3p expression alters the viability of CRC cells following treatment with chemotherapy agents.** Viability of LOVO cells transfected with a lentiviral vector carrying a miR-365a-3p-specific mimic and treated with increasing doses of **(A)** doxorubicin, **(B)** fluorouracil or **(C)** cisplatin. Viability of SW480 cells transfected with a lentiviral vector carrying a miR-365a-3p-specific inhibitor and treated with increasing doses of **(D)** doxorubicin, **(E)** fluorouracil or **(F)** cisplatin. miR, microRNA. **P<0.05 compared with group LV-INC or LV-mNC. *** P<0.01 compared with group LV-INC or LV-mNCFigure 3. Interference of miR-365a-3p expression alters the viability of CRC cells following treatment with chemotherapy agents. Viability of LOVO cells transfected with a lentiviral vector carrying a miR-365a-3p-specific mimic and treated with increasing doses of **(A)** doxorubicin, **(B)** fluorouracil or **(C)** cisplatin. Viability of SW480 cells transfected with a lentiviral vector carrying a miR-365a-3p-specific inhibitor and treated with increasing doses of **(D)** doxorubicin, **(E)** fluorouracil or **(F)** cisplatin. miR, microRNA. **P<0.05 compared with group LV-INC or LV-mNC. *** P<0.01 compared with group LV-INC or LV-mNC.

To investigate the effect of miR-365a-3p modulation on CRC cell survival, a cellular viability assay was conducted using LOVO and SW480 cell lines transfected with a lentiviral vector carrying a miR-365a-3p-specific mimic or inhibitor, respectively, and treated with increasing doses of doxorubicin, fluorouracil or cisplatin. The results revealed that the viability of LOVO cells transfected with miR-365a-3p mimic was significantly suppressed compared with control group (Fig. 3A-C). Conversely, the viability was significantly increased in SW480 cells transfected with miR-365a-3p inhibitor vector following treatment with the three chemotherapy agents (Fig. 3D-F).

### KLF3 is a target gene of miR-365a-3p and affects chemoresistance in CRC cell lines

**Figure 4:**
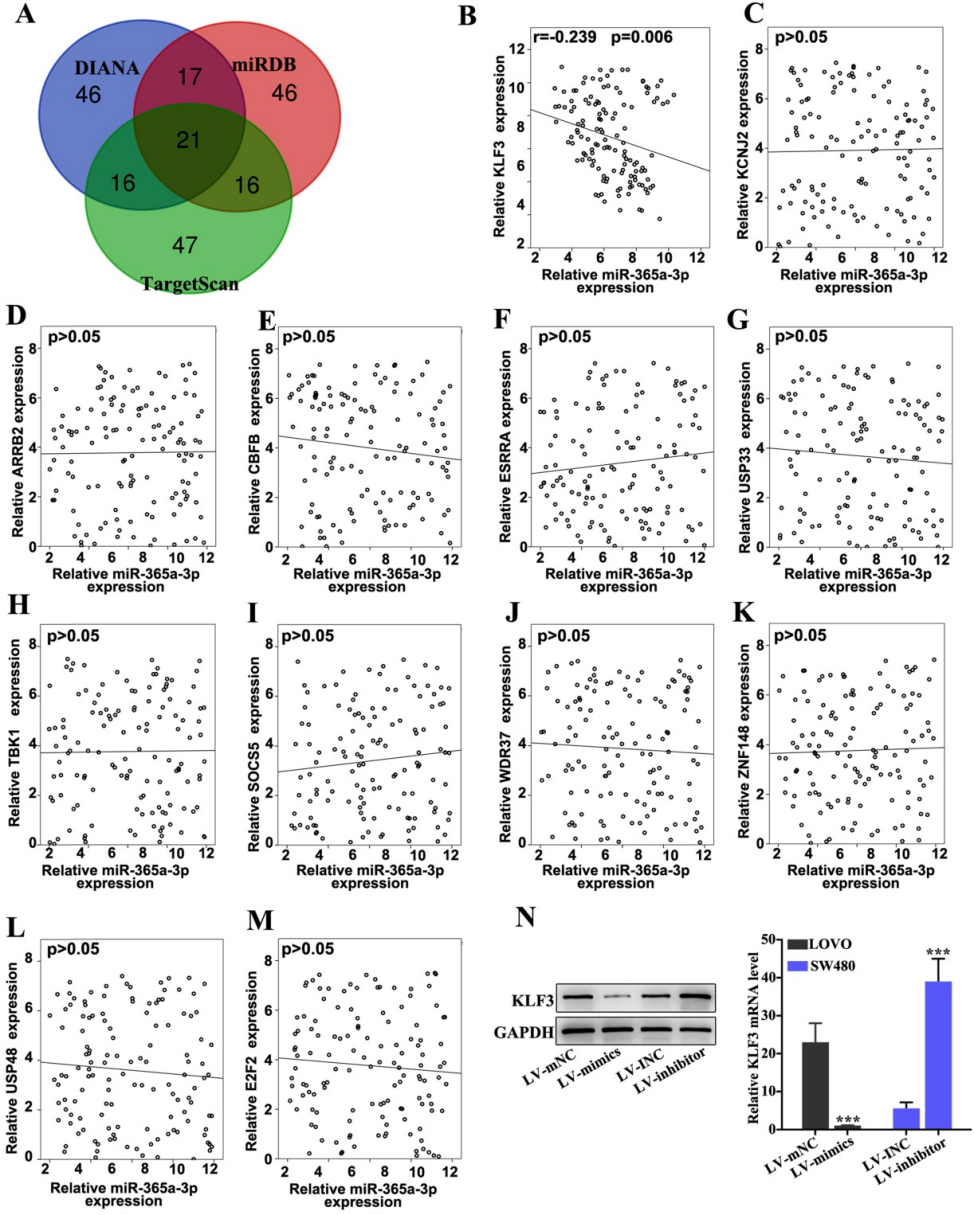
** The target gene of miR-365a-3p was predicted via bioinformatics analysis. (A)** miR-365a-3p target genes were predicted using three miRNA databases (TargetScan, miRDB and DIANA tools). The top 100 candidate genes from each database were used for subsequent analysis **(B-M)** RT-qPCR was used to determine the association between miR-365a-3p expression and mRNA expression levels of the top ranked candidate target genes, including ARRB2, CBFB, ESRRA, USP33, TBK1, SOCS5, WDR37, ZNF148, USP48 and E2F2. **(N)** Western blotting and RT-qPCR analysis of protein and mRNA expression levels of KLF3, respectively, in LOVO and SW480 cells transfected with a lentiviral vector carrying a miR-365a-3p-specific mimic or inhibitor, respectively. miR/miRNA, microRNA; RT-qPCR, reverse transcription-quantitative PCR; KLF3, Kruppel-like factor 3; ARRB2, arrestin β2; CBFB, core-binding factor subunit β; ESRRA, estrogen related receptor α; USP33, ubiquitin specific peptidase 33; TBK1, TANK binding kinase 1; SOCS5, suppressor of cytokine signaling 5; WDR37, WD repeat domain 37; ZNF148, zinc finger protein 148; USP48, ubiquitin specific peptidase 48; E2F2, E2F transcription factor 2. **P<0.05 compared with group LV-INC or LV-mNC. *** P<0.01 compared with group LV-INC or LV-mNC.

The target gene of miR-365a-3p was predicted via bioinformatics analysis using three public miRNA databases, miRDB, DIANA tools and TargetScan (Fig. 4A). The expression levels of top 12 genes predicted by all three databases were further analyzed in CRC samples using RT-qPCR (Fig. 4B-M). Using linear regression analysis, it was revealed that only the expression levels of KLF3 were significantly negatively correlated with miR-365a-3p expression in CRC samples (Fig. 4B). To further validate these findings, the effect of miR-365a-3p modulation on KLF3 mRNA and protein expression levels was determined. The results demonstrated that the expression levels of KLF3 were significantly downregulated in LOVO cells transfected with the miR-365a-3p mimic vector, while the expression levels of KLF3 were significantly upregulated in SW480 cells transfected with the miR-365a-3p inhibitor vector (Fig. 4N).

**Figure 5:**
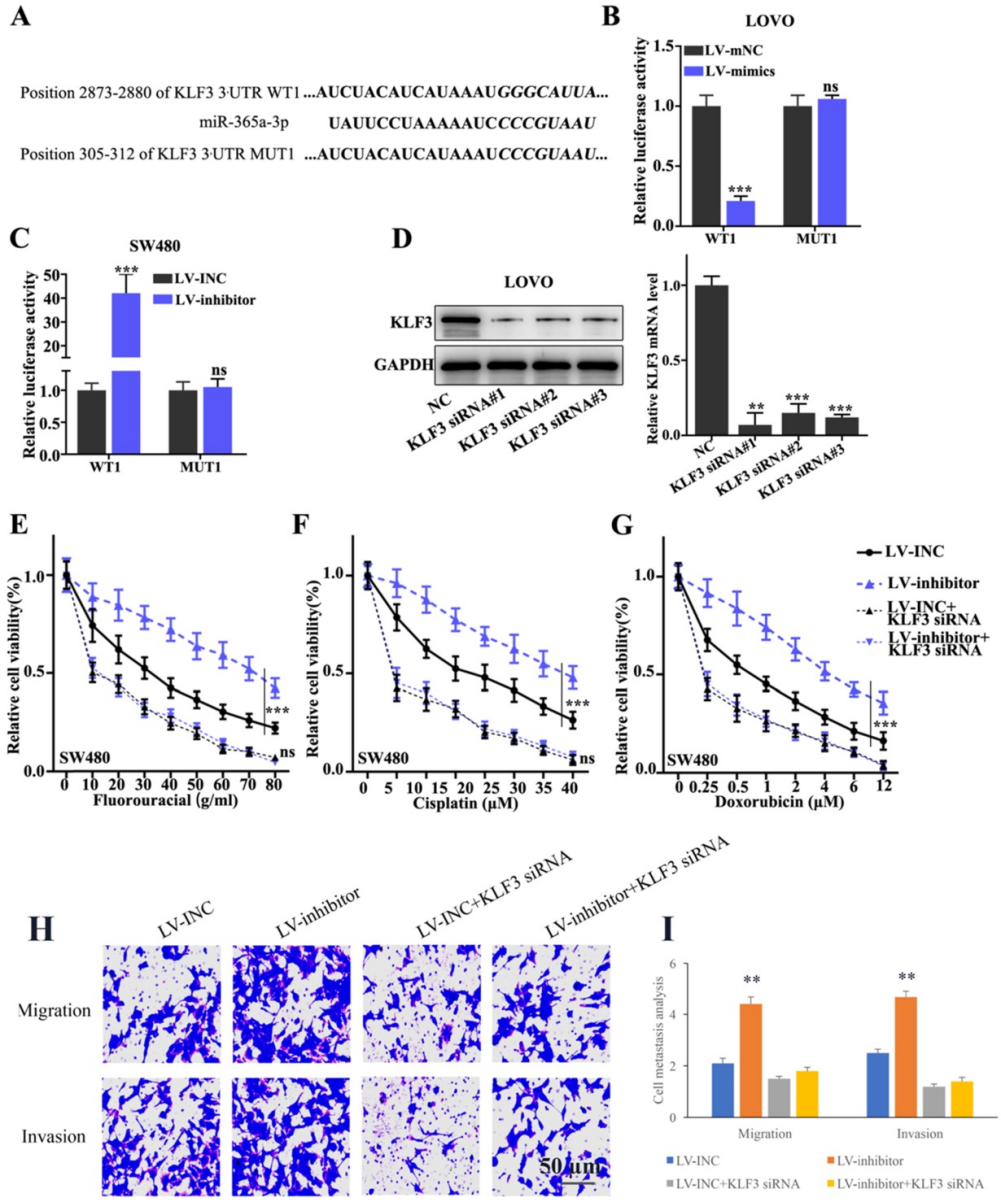
** KLF3 is a target gene of miR-365a-3p and modulates chemoresistance in CRC cell lines. (A)** miR-365a-3p binding site in the WT-KLF3 3'-UTR region was predicted and vectors carrying WT- and MUT-KLF3 3'-UTR region were synthesized. **(B and C)** Dual luciferase reporter gene assay was conducted to investigate the effect on KLF3 expression in LOVO and SW480 cells co-transfected with the lentiviral vectors carrying a miR-365a-3p-specific inhibitor or mimic, respectively, and WT- or MUT-KLF3 3'-UTR vectors. **(D)** Western blotting and reverse transcription-quantitative PCR were used to determine the regulatory effects of KLF3-specific siRNAs on KLF3 protein and mRNA expression levels in LOVO cells, respectively. **(E-G)** Viability of SW480 cells transfected with a lentiviral vector carrying a miR-365a-3p-specific inhibitor, with or without the co-transfection with KLF3-specific siRNAs. Each group of cells was treated with increasing doses of fluorouracil, cisplatin or doxorubicin. **(H and I)** Cell migration and invasion were measured following transfection with a lentiviral vector carrying a miR-365a-3p-specific inhibitor, with or without co-transfection with KLF3-specific siRNAs. miR, microRNA; WT, wild-type; MUT, mutant; KLF3, Kruppel-like factor; UTR, untranslated region; siRNA, small interfering RNA. **P<0.05 compared with group NC, LV-mNC, or LV-INC. *** P<0.01 compared with group NC, LV-mNC, or LV-INC.

In addition, to validate the interaction between miR-365a-3p and KLF3, the binding site for miR-365a-3p within KLF3 3'-UTR region was predicted. Subsequently, vectors carrying WT- and MUT- KLF3 3'-UTR sequences were constructed (Fig. 5A) and a dual luciferase reporter gene assay was used to evaluate the effects of miR-365a-3p mimic on the relative luciferase activity of LOVO cells transfected with vectors carrying the WT- or MUT-KLF3 3'-UTR region. The results revealed that the relative luciferase activity was significantly decreased in LOVO cells transfected with the lentiviral vector carrying the miR-365a-3p mimic and WT-KLF3 3'-UTR, while no significant differences were observed in the cells transfected with the MUT-KLF3 3'-UTR (Fig. 5B). In addition, the relative luciferase activity was significantly increased in SW480 cells transfected with the lentiviral vector carrying the miR-365a-3p inhibitor and WT-KLF3 3'-UTR (Fig. 5C). To investigate the effect of KLF3 on CRC cell lines, KLF3-specific small interfering RNAs (siRNAs) were transfected into cells and the knockdown efficiency of KLF3 was determined using RT-qPCR and western blotting (Fig. 5D). Cell viability was measured to evaluate the effects of the co-transfection of KLF3 siRNA and miR-365a-3p on the chemoresistance of SW480 cells. The results discovered that chemoresistance was enhanced in cells transfected with the vector carrying the miR-365a-3p inhibitor following treatment with chemotherapy agents, fluorouracil, cisplatin or doxorubicin, while the co-transfection with KLF3 siRNA abrogated the chemoresistance of SW480 cells (Fig. 5E-G). In addition, the migratory and invasive abilities of cells transfected with the vector carrying miR-365a-3p inhibitor were significantly increased. However, the co-transfection with KLF3 siRNA markedly suppressed the migratory and invasive abilities of cells (Fig. 5H and I).

## Discussion

Despite the advances in surgical treatment for patients with CRC, metastasis remains a crucial factor for predicting overall prognosis 20. Therefore, identifying more sensitive markers for the early detection of metastatic tumors and being able to more effectively control malignant lesions are of great clinical importance for CRC treatment.

Previous studies have identified several molecular biomarkers which may provide promising predictive potential. For example, the presence of microsatellite instability-low sites combined with microsatellite alterations at selected tetranucleotide repeats was found to be associated with cancer metastasis and disease prognosis 21. Furthermore, miR-302a was discovered to be associated with increased metastatic activity and cetuximab resistance in cancer cells by targeting nuclear factor I B and CD44 22. In addition, exosomes secreted by cancer-associated-fibroblasts enhanced tumor cell proliferation and chemoresistance by upregulating miR-92a-3p expression levels 23. These findings indicated that miRNAs may be sensitive biomarkers and potential regulators of cancer progression and metastasis.

However, the significance of miR-365a-3p in CRC remains unclear. The results of present study demonstrated, for the first time, that the expression levels of miR-365-3p were significantly downregulated in CRC tissues and cell lines. In addition, the overexpression of miR-365-3p suppressed CRC cells, while the knockdown of miR-365-3p promoted them. The study also identified a direct binding site between miR-365-3p and KLF3, and miR-365-3p was found to negatively regulate KLF3 expression. Notably, the dysregulation of miR-365-3p/KLF3 axis was associated with migratory, invasive and chemoresistant abilities of CRC cells. These findings indicated that miR-365-3p may serve as a potential biomarker and target for CRC treatment.

Multiple previous studies have demonstrated that miR-365a-3p exerted inhibitory effects on tumor metastasis in numerous types of malignant disease 24, 25. For example, one study revealed that miR-365a-3p significantly suppressed liver cancer cell proliferation, migration and invasion via inhibition of ten-eleven translocation-1, which was identified as a downstream target of miR-365a-3p 16. Another study demonstrated that miR-365a-3p suppressed pancreatic ductal adenocarcinoma cell viability, migration and clonogenicity by downregulating NF-κB and REL protooncogene, NF-κB subunit 26. miR-365a-3p also exerted suppressive effects in CRC by inhibiting ADAM metallopeptidase domain 10 expression and the associated JAK/STAT signaling 24. However, another study reported that miR-365a-3p may serve as an oncogene, as miR-365-3p increased laryngeal squamous cell carcinoma cell proliferation by targeting PI3K/AKT signaling pathway via inhibiting phosphorylation of AKT on serine 473 25. The results from the present study provided a novel insight into the complex and diversified role of miR-365-3p in various cancer types. However, further investigations are required to determine the effects of miR-365-3p in CRC cells.

Nonetheless, the present study has some limitations. First, the study was based on *in vitro* CRC cell line studies and cancer samples obtained from a single clinical center. Therefore, multi-centered clinical trials are required to further validate the current findings. Second, the expression levels of miR-365a-3p and the complex regulatory network between miR-365a-3p and its target gene signaling pathways in CRC were not fully elucidated. Thus, determining potential novel therapeutic strategies for CRC that target miR-365a-3p require future investigation.

In conclusion, the present study demonstrated for the first time that miR-365a-3p expression levels were significantly downregulated in CRC tissues, especially in patients with lymph node and distant organ metastases. Further experiments revealed that miR-365a-3p suppressed the migration, invasion and chemoresistance of tumor cells by inhibiting KLF3. These findings provided a novel insight into the tumor-suppressive role of miR-365a-3p in CRC, and suggests a new target for CRC treatment.

## Figures and Tables

**Table 1:** Clinical and pathological features of patients

Features	Number of patients
**Age**	
≤50	41
>50	59
**Sex**	
Male	62
Female	38
**Lymph node status**	
N0	55
N1	39
N2	6
**Tumor stage**	
I-II	79
III-IV	21
